# Novel HDAd/EBV Reprogramming Vector and Highly Efficient Ad/CRISPR-Cas Sickle Cell Disease Gene Correction

**DOI:** 10.1038/srep30422

**Published:** 2016-07-27

**Authors:** Chao Li, Lei Ding, Chiao-Wang Sun, Li-Chen Wu, Dewang Zhou, Kevin M. Pawlik, Alireza Khodadadi-Jamayran, Erik Westin, Frederick D. Goldman, Tim M. Townes

**Affiliations:** 1 Department of Biochemistry and Molecular Genetics, School of Medicine, University of Alabama at Birmingham, 1720 2nd Ave South, Birmingham, AL 35294, USA; 2 UAB Stem Cell Institute, School of Medicine, University of Alabama at Birmingham, 1720 2nd Ave South, Birmingham, AL 35294, USA; 3 Department of Pediatrics, Division of Hematology/Oncology, School of Medicine, University of Alabama at Birmingham, 1720 2nd Ave South, Birmingham, AL 35294, USA

## Abstract

CRISPR/Cas enhanced correction of the sickle cell disease (SCD) genetic defect in patient-specific induced Pluripotent Stem Cells (iPSCs) provides a potential gene therapy for this debilitating disease. An advantage of this approach is that corrected iPSCs that are free of off-target modifications can be identified before differentiating the cells into hematopoietic progenitors for transplantation. In order for this approach to be practical, iPSC generation must be rapid and efficient. Therefore, we developed a novel helper-dependent adenovirus/Epstein-Barr virus (HDAd/EBV) hybrid reprogramming vector, rCLAE-R6, that delivers six reprogramming factors episomally. HDAd/EBV transduction of keratinocytes from SCD patients resulted in footprint-free iPSCs with high efficiency. Subsequently, the sickle mutation was corrected by delivering CRISPR/Cas9 with adenovirus followed by nucleoporation with a 70 nt single-stranded oligodeoxynucleotide (ssODN) correction template. Correction efficiencies of up to 67.9% (β^A^/[β^S^+β^A^]) were obtained. Whole-genome sequencing (WGS) of corrected iPSC lines demonstrated no CRISPR/Cas modifications in 1467 potential off-target sites and no modifications in tumor suppressor genes or other genes associated with pathologies. These results demonstrate that adenoviral delivery of reprogramming factors and CRISPR/Cas provides a rapid and efficient method of deriving gene-corrected, patient-specific iPSCs for therapeutic applications.

Sickle Cell Disease (SCD) is a devastating inherited disorder resulting from a single DNA base mutation (A>T) in the sixth codon of the β-globin gene^1^,^2^. Currently, the only available cure is allogeneic bone marrow transplantation, which is limited to a minority of patients with an available histocompatible donor^3^. Correction of the sickle mutation in autologous hematopoietic stem/progenitor cells would provide a therapy available to all patients.

Following the seminal discovery of induced Pluripotent Stem Cells (iPSCs) by Takahashi and Yamanaka^4^, iPSCs have been used extensively as a tool for studying normal development and for developing new strategies for regenerative medicine and patient-specific cell therapy. Episomal reprogramming, which was first reported by the Thomson laboratory^5^ and later improved by the Yamanaka laboratory^6^,^7^, provides a method that avoids random chromosomal integration of exogenous reprogramming factors. However, these methods rely on electroporation of 3–4 plasmids to deliver 6–7 factors into target cells. Electroporation of multiple plasmids is toxic to cells and results in reprogramming at low efficiency^8^. Therefore, we developed a helper-dependent adenovirus/Epstein-Barr virus (HDAd/EBV) episomal vector that is capable of delivering a large number of reprogrammimg factors to somatic cells with high efficiency and reprograms these cells with rapid kinetics.

The rapid kinetics of reprogramming after somatic cell nuclear transfer (SCNT) suggest that synergistic effects of a large number of factors are important in the efficiency and rapidity of reprogramming^9^,^10^,^11^,^12^. A number of factors that improve reprogramming efficiency or kinetics^13^,^14^,^15^,^16^,^17^,^18^,^19^,^20^,^21^ have been reported; however, most of these factors have not been generally used in the field due to the size limitation of non-integrating vectors. The 6-factor HDAd/EBV vector that we report in this paper provides a foundation for production of additional vectors containing ten or more reprogramming factors that increase the efficiency and rapidity of iPSC production.

Targeted correction of the sickle mutation in SCD patient-derived iPSCs has recently been reported by several groups using HDAdV^22^, ZFNs^23^,^24^,^25^, TALENs^25^ and CRISPR/Cas^26^. However, in all of these cases, correction efficiencies were relatively low, and the methods relied on selection of targeted cells with antibiotics followed by time-consuming removal of the selection markers. Sib-selection without antibiotics has been used to isolate human iPSCs with edited endogenous genes^27^; however, three or more rounds of sib-selection were required to isolate single colonies of corrected cells. Since each round of sib-selection requires 8–15 days to complete, correction of the sickle mutation by this method is laborious, time-consuming and expensive. In this paper, we report the development of an efficient, rapid, and scarless CRISPR/Cas method to correct the sickle mutation in patient-derived iPSCs.

## Results

### Derivation of SCD Patient-Specific iPSC Lines with a 6-Factor HDAd/EBV Hybrid Reprogramming Vector

**Figure 1:**
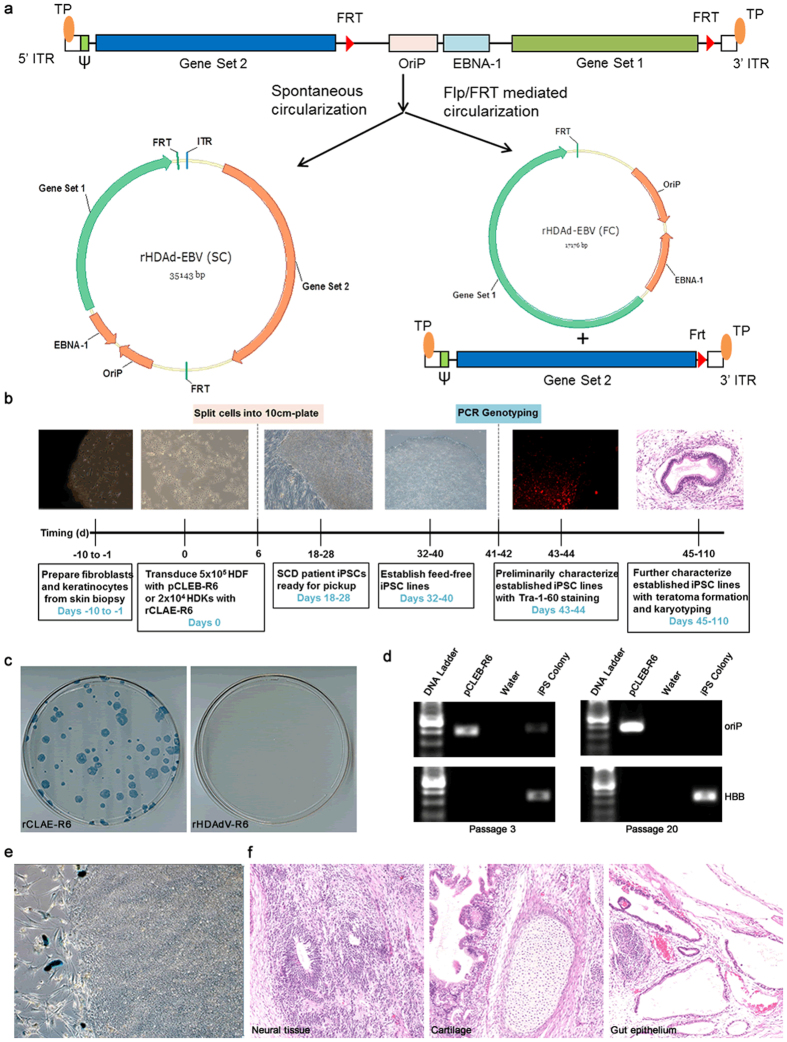
SCD patient-specific iPSCs derived with a novel 6-factor HDAd/EBV hybrid reprogramming vector. (**a**) Schematic representation of two circularization strategies for HDAd/EBV hybrid vectors in transduced cells to produce episomes. The vector is efficiently circularized by recombinase systems, e.g. Flp/FRT. In the absence of recombinase, a small but significant percentage of HDAd/EBV vectors spontaneously circularize in transduced cells. (**b**) Timeline of SCD patient fibroblast reprogramming with pCLEB-R6 or keratinocytes with rCLAE-R6. (**c**) Alkaline phosphatase (AP) staining of reprogrammed SCD keratinocytes. Twenty-four days post-transduction with rCLAE-R6 (left) and rHDAdV-R6 (right). (**d**) PCR amplification of oriP in passage 3 (left) and passage 20 (right) SCD iPSCs to detect residual rCLAE-R6. We analyzed six colonies, and all six were free of rCLAE-R6 after passage 20. Only one iPS colony is shown in Fig. 1d (last lane in each panel). OriP/EBNA1 vectors replicate only once per cell cycle; therefore, in the absence of selection, episomes are lost at a rate of approximately 5% per cell generation due to defects in plasmid replication and partitioning. The second lane in each panel (pCLEB-R6) is a PCR control using untranduced plasmid DNA. (**e**)X-Gal staining of reprogrammed SCD keratinocytes 24 days post-transduction with rCLAE-R6, which contains LacZ. LacZ was only expressed in parental keratinocytes and not in iPSC clones. (**f**) Hematoxylin and eosin staining of teratoma sections derived from SCD iPSCs.

HDAdV vectors are derived from adenoviruses and all viral coding regions have been removed^28^,^29^. The removal of viral genes not only creates 37 kb of space for foreign DNA but also renders these vectors far less cytotoxic and immunogenic than previous generations of adenoviral vectors^30^,^31^,^32^. Although HDAd vectors are advantageous for many reasons, they are not suitable for reprogramming due to their transient expression nature. However, HDAd/EBV hybrid vectors, which are derived by incorporating oriP/EBNA1 components of EBV into HDAdV, provide longer-lasting gene expression once circularized either spontaneously^33^ or by recombinase systems including Flp/FRT and Cre/LoxP^33^,^34^,^35^,^36^, in transduced cells^33^,^34^ and *in vivo*^35^,^36^ (Fig. 1a). Circularization of HDAd/EBV hybrid vectors after transduction of primary somatic cells provides a potentially ideal non-integrating reprogramming vector approach.

We constructed an HDAd/EBV reprogramming hybrid vector, pCLAE-R6, as illustrated in Supplementary Fig. 1a. The hybrid vector contains an EF1α promoter driving expression of a polycistronic Oct4-2A-Sox2-2A-Klf4 sequence, which we published previously^37^, plus 3 additional sequences (L-Myc, Lin-28, and p53shRNA) that augment reprogramming^6^. To verify the functionality of this factor combination and configuration as well as the oriP/EBNA1 components, the intermediate 6-factor episomal vector, pCLEB-R6, was used to reprogram SCD patient fibroblasts. Although the reprogramming efficiency by electroporation of plasmid pCLEB-R6 was higher than with previously published 6-factor episomal vectors^6^, we were not able to induce iPSCs from all donors unless the culture medium was supplemented with sodium butyrate^38^ (data not shown). Therefore, we constructed pCLAE-R6 by inserting a 17 kb stuffer containing LacZ into pCLEB-R6 and packaging this large piece of DNA into an HDAd/EBV hybrid viral vector, rCLAE-R6 (Supplementary Fig. 1a,c). rCLAE-R6 transduced both mouse and human fibroblasts with high efficiency and with minimal cell death compared to electroporation of pCLAE-R6 (Supplementary Fig. 2a). Human keratinocytes, which are reprogrammed with higher efficiency and kinetics^39^ than fibroblasts, are easily damaged by electroporation of large plasmids. However, we demonstrate that human keratinocytes can be transduced by rCLAE-R6 at high efficiency with low MOI (Supplementary Fig. 2b). Starting with 2 × 10^4^ initial keratinocytes, iPSC colonies were consistently generated by rCLAE-R6 transduction without using sodium butyrate (Fig. 1b,c). Parallel reprogramming of keratinocytes was performed with rHDAdV-R6, which contains the same six reprogramming factors as rCLAE-R6 but does not contain oriP/EBNA1. This vector failed to generate iPSC colonies (Fig. 1c). These data suggest that the high reprogramming efficiency of rCLAE-R6 results from episomal replication of the vector and persistent expression of the six factors.

In passage 3 iPSCs, the absence of X-Gal staining and the presence of oriP by PCR amplification suggest that expression of the transgenes is silenced (Fig. 1d,e). In passage 20 iPSCs, oriP is undetectable by PCR (Fig. 1d); therefore, rCLAE-R6 is lost spontaneously during cell division. Over 90% of the iPSC lines generated with rCLAE-R6 formed tissues derived from all three germ layers when these iPSCs were transplanted under the kidney capsules of immunodeficient (NSG) mice (Fig. 1f). The karyotypes of three clones were analyzed and all three were normal (Supplementary Fig. 2c).

### Rapid Isolation of Scarless, Homozygously Corrected SCD Patient iPSCs

To pursue a CRISPR/Cas9-enhanced sickle gene correction approach, ten single guide RNA (sgRNA) expression vectors were produced and tested for specific DNA cleavage after transfection into 293A cells. The five sgRNAs that generated indels with highest efficiency were used for gene correction experiments (Supplementary Fig. 3a,b). Different combinations of Cas9 and sgRNAs, including wild type Cas9 (wtCas9) + single sgRNA, D10A Cas9 nickase (nCas9) + single sgRNA, and nCas9 + double sgRNAs^40^,^41^ were tested for correction efficiency and fidelity in SCD iPSCs using traditional antibiotic selection based methods (Supplementary Fig. 4). Targeting results indicated the following: (1) correction efficiencies with nCas9 + single sgRNA T1 were very low; (2) correction efficiencies with wtCas9 + T1 were highest among the Cas9 + gRNA combinations, but the majority of corrected clones contained a mutation near the protospacer [This may be due to indels created by retargeting. Retargeting occurs when Cas9 RNPs (Cas9/gRNA complexes), which are still present in the cell after HDR, recognize and recleave the DNA. The T1 sgRNA does not contain the sickle mutation and, therefore, Cas9/T1sgRNA cleaves uncorrected and corrected alleles equivalently. This phenomenon has been reported by many investigators including Hoban *et al*.^42^]; (3) correction efficiencies with nCas9 + (T8 + T2) and wtCas9 + T2 were medium to high with all corrected iPSC clones free of mutations near the protospacer. Interestingly, homozygously corrected iPSC clones were obtained only with wtCas9 + T2 (Supplementary Fig. 4e; Supplementary Table 1). We suspect that all iPSC clones corrected with wtCas9 + T2 were free of mutations because the T2 sgRNA specifically targets the β^S^ allele; therefore, after correction of the mutation, the T2 sgRNA does not efficiently recognize the β^A^ allele and retargeting does not occur.

**Figure 2:**
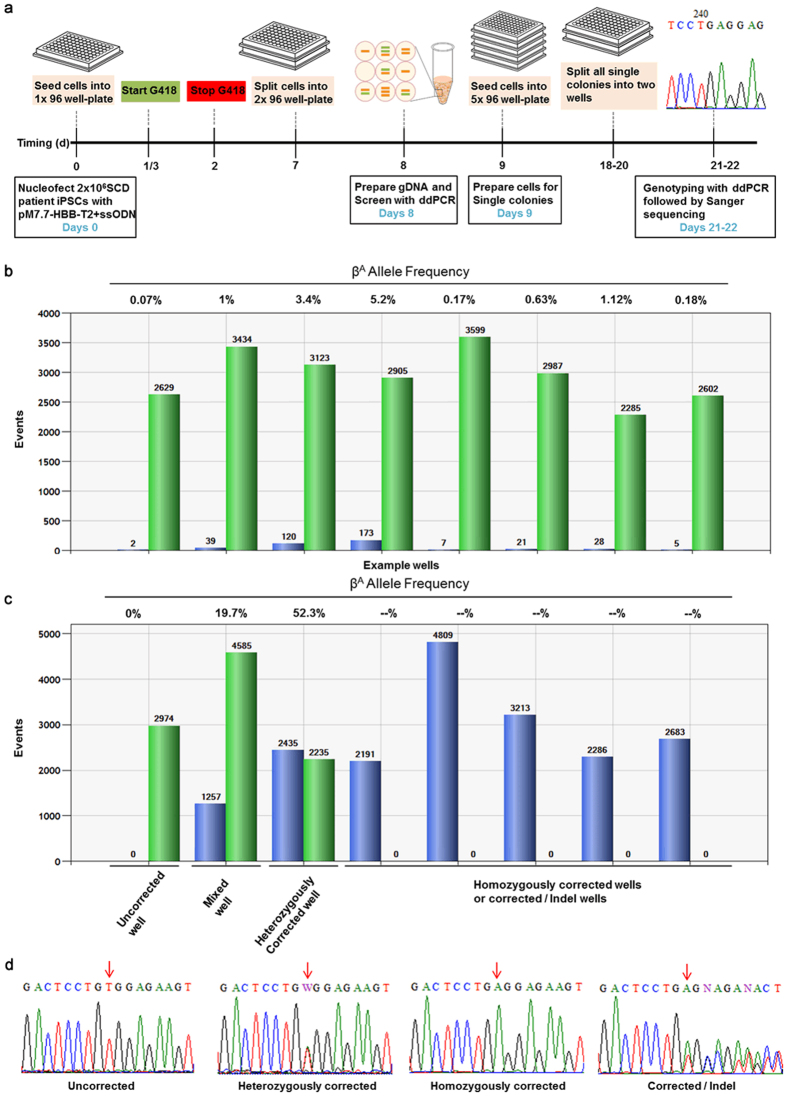
Rapid isolation of homozygously corrected SCD patient-derived iPSCs by sib-selection. (**a**) Schematic representation of the strategy to rapidly isolate corrected iPSCs. (**b**) *HBB* corrected allele frequency as measured by ddPCR after the first sib-selection. Green bar: β^S^-positive droplets; Blue bar: β^A^-positive droplets. (**c**) *HBB* corrected allele frequency as measured by ddPCR after the second sib-selection. Green bar: β^S^-positive droplets; Blue bar: β^A^-positive droplets. (**d**) Representative Sanger sequencing results of PCR amplicons generated from an uncorrected SCD iPSC line, a heterozygously corrected line, a homozygously corrected line, and a singly-corrected allele/indel line.

We also used the wtCas9 + T2 combination for correction of SCD iPSCs with a 70 nt ssODN. The T2 sgRNA guided Cas9 to cleave the β^S^ allele (GTG) just one base pair downstream of the sickle (T) mutation and the 70 nt ssODN contained the wild-type β^A^ sequence (GAG) in the middle of the oligo. Our initial allelic correction frequencies were low (0.1–0.3%; data not shown); therefore, three rounds of sib-selection were required to isolate corrected clones. In order to expedite the sib-selection process, we added a temporary G418 selection step after electroporation to enrich for transduced cells (Fig. 2a). The wtCas9 gene, which was driven by a Cbh promoter, and the T2 sgRNA sequence, which was driven by the hU6 promoter, were transferred to pM7.7 containing a neomycin resistance gene expression cassette. After transfection with pM7.7/wtCas9/T2 plus the 70 nt correction ssODN, the cells were seeded into 96-well plates and cultured for 40 hr with G418. DNA was extracted from three-fourths of the cells after reaching confluency and analyzed by droplet digital PCR (ddPCR; Bio Rad) for β^A^ (corrected) and β^S^ alleles. Wells with a β^A^ allele frequency above 5% were replated and corrected single clones were isolated in the second round of sib-selection (Fig. 2b–d). Surprisingly, over 50% of clones containing a β^A^ allele were homozygous for the corrected allele (β^A^/β^A^). In two additional experiments, homozygously corrected clones were also isolated from iPSCs derived from two different patients.

**Figure 3:**
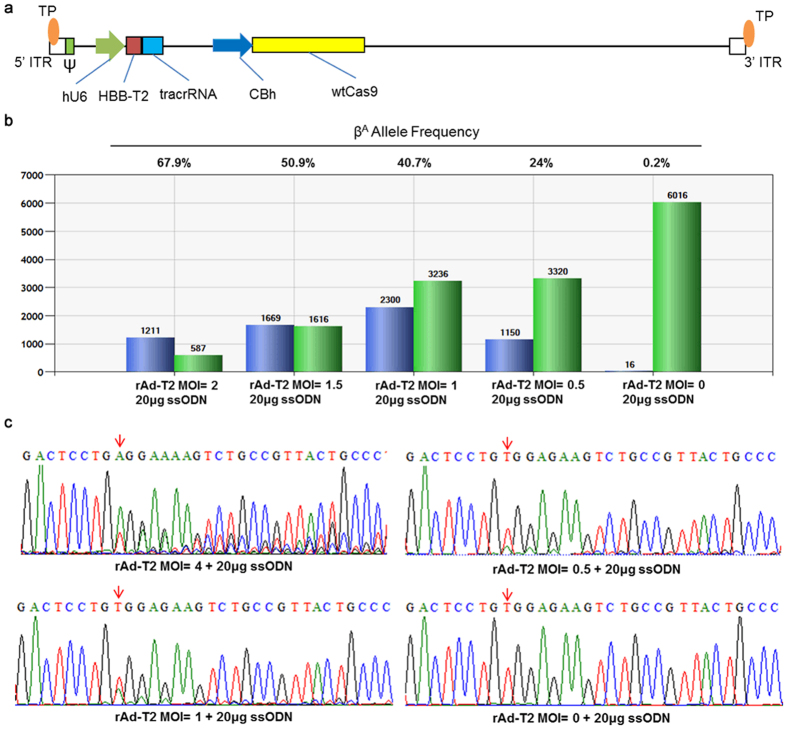
Improved targeting efficiency with a CRISPR/Cas-expressing adenoviral vector. (**a**) Schematic representation of the first generation adenoviral vector used to express CRISPR/Cas. (**b**) *HBB* corrected allele frequency as measured by ddPCR in pooled SCD iPSCs at 72 hr post-transduction of rAd-T2 + ssODN. Green bar: β^S^-positive droplets; Blue bar: β^A^-positive droplets. (**c**) Sanger sequencing results of PCR amplicons generated from pooled SCD iPSC lines transduced with rAd-T2 + ssODN.

Although the methods described above provide a relatively rapid protocol for isolating corrected iPSCs, the procedure could be even faster if correction efficiencies were high enough to allow the isolation of corrected colonies after only one round of sib-selection. Unfortunately, the large size of the pM7.7/wtCas9/T2 plasmid (over 8 kb) severely limits the molar amount of DNA that can be electroporated into the cells, and this limits the correction efficiency (Supplementary Fig. 4c,d). Also, electroporation of large plasmids is toxic to cells. In order to deliver the CRISPR/Cas system into iPSCs with high efficiency and low toxicity, we constructed and packaged a first generation adenoviral vector, rAd/wtCas9/T2, for delivering wtCas9 and T2 sgRNA (Fig. 3a). By infecting SCD iPSCs with rAd/wtCas9/T2 one hour prior to electroporating 20 μg of a 70 nt ssODN correction template, β^A^ allele frequencies (β^A^/[β^A^+β^S^]) of up to 67.9% were detected by ddPCR and verified by Sanger sequencing (Fig. 3b,c; Supplementary Fig. 6c). Although higher correction efficiencies were achieved if MOIs greater than 1.0 were used, the rate of indel formation increased to unacceptable levels (Fig. 3c). Therefore, per our optimized protocol, we infected 5 × 10^5^ SCD iPSCs with rAd/wtCas9/T2 at an MOI of 1.0 and, subsequently, electorporated the cells with 20 μg of ssODN. This procedure resulted in the consistent isolation of homozygously corrected iPSC clones after one round of sib-selection.

The potential for off-target modifications has always been a concern for engineered nuclease-assisted gene editing approaches including CRISPR/Cas9. Relatively high levels of off-target mutagenesis by Cas9-gRNAs in cancer cell lines have been described^43^. However, WGS studies by other groups showed that off-target modifications are rare in human iPSCs and ESCs^44^,^45^.

To assess the specificity of our procedure, WGS of SCD iPSCs, before and after CRISPR/Cas9-enhanced gene targeting, was performed. Two wtCas9 + T2+ssODN homozygously corrected clones, one from each of two patients, were sequenced. Potential off-target sites were identified by aligning the CRISPR/Cas9 guide sequence to the hg19 reference genome using EMBOSS fuzznuc software (v6.6.0.0)^46^ and allowing for a maximum of five mismatches (Supplementary Fig. 7). These sites were then analyzed for variations in the corrected iPSC samples, and no mutations (SNVs nor indels) were detected in 1467 potential off-target sites (Table 1). Furthermore, the WGS data was screened for known associations with disease in the databases ClinVar, GWAS Catalogue, and COSMIC, and no disease-causing variants in sequences with or without homology to the gRNA were observed.

## Discussion

In this study, we developed a novel hybrid HDAd/EBV reprogramming vector and proved the feasibility of using this vector to convert human SCD patient keratinocytes to iPSCs with high efficiency. Reprogramming was achieved by the efficient delivery of six factors (rCLAE-R6) but additional factors can be added by replacing the 17 kb of stuffer DNA that was inserted to allow packaging. Up to 37 kb of foreign DNA can be cloned into the vector and replicated autonomously after spontaneous formation of the episome. A large number of reprogramming factors has been reported and many of these factors function synergistically to increase reprogramming efficiency and kinetics^13^,^14^,^15^,^16^,^17^,^18^,^19^,^20^,^21^. Therefore, our novel HDAd/EBV vector provides an important tool to define the optimal combination of factors that produce the highest quality iPSCs. Also, the high transduction efficiency of rCLAE-R6 and low toxicity to target cells permits the formation of iPSCs from a relatively small population of primary cells. This is important because human iPSCs for therapeutic applications must be obtained from patient-specific tissue biopsies. Although spontaneous circularization is easy and sufficient to obtain high quality iPSCs from a relatively small population of primary cells, we plan to increase efficiency further by including an inducible recombinase expression cassette in rCLAE-R6^33^,^34^,^35^,^36^.

In this study, we also developed a rapid protocol for isolating CRISPR/Cas corrected SCD iPSCs by sib-selection. By combining the use of an adenoviral vector for CRISPR/Cas delivery with electroporation of an ssODN correction template, we are able to achieve β^A^ allele frequencies (β^A^/[β^A^+β^S^]) of up to 67.9% and to isolate homozygously corrected iPSCs in a single round of sib-selection. Two other groups have used first generation adenoviral vectors^47^ or HDAdV^48^ to deliver CRISPR/Cas and correction templates into cells. Although the generation of double strand breaks (DSBs) was high in both reports, HDR was inefficient because the concentration of correction template was low. In our protocol, a high concentration of ssODN correction template is delivered by electroporation one hour after transient transduction of the cells with rAd/wtCas9/sgRNA. Efficient generation of DSBs in the presence of high concentrations of ssODN results in highly efficient gene correction by HDR. In addition, WGS of corrected iPSC lines demonstrated no CRISPR/Cas modifications in 1467 potential off-target sites and no modifications in tumor suppressor genes or other genes associated with pathologies. These results demonstrate that adenoviral delivery of reprogramming factors and CRISPR/Cas provides a rapid and efficient method for deriving gene-corrected, patient-specific iPSCs for therapeutic applications.

## Methods

### Patient Information

All methods were carried out in accordance with approved guidelines. All experimental protocols were approved by the UAB Institutional Review Board (IRB). Skin biopsies were obtained in the Dermatology Clinic after informed consent and cultured initially by personnel in the Department of Dermatology. At passage two, the cultures were transferred to our laboratory.

### Reprogramming Vector Construction

A detailed construction strategy is illustrated in Supplementary Fig. 1a.

A CLEB-B4 miniGene was synthesized by Integrated DNA Technologies (IDT) and inserted into pCLEB to produce the backbone vector pCLEB-B4. The CMV expression cassette was removed from pCEP4 to make pCEP4.2, and then an oriP-EBNA1 fragment was transferred from pCEP4.2 to pCLEB-B4 to generate pCLEB-EBV.

OCT3/4-P2A-SOX2-P2A-KLF4 was transferred from pPK332^37^ to the shuttle vector pM7.5-EF1α to produce pM7.5E-OSK and, subsequently, the woodchuck hepatitis post-transcriptional regulatory element (WPRE) was inserted upstream of the polyadenylation signal to produce pM7.5E-OSKw.

Shp53 from pCXLE-hOCT3/4-shp53-F (Addgene: plasmid #27077) and hUL from pCXLE-hUL (Addgene: plasmid #27080) were transferred sequentially to the shuttle vector pM.3-CAG to produce pM7.3Cgh-shp53-hUL, which was combined with elements of pM7.5E-OSKw to result in pM7-R6.

The six reprogramming factors were then transferred from pM7-R6 to pCLEB-EBV to obtain pCLEB-R6.

A 17 kb stuffer DNA fragment was inserted into pCLEB-R6 to generate pCLAE-R6.

### Derivation and Characterization of Human iPSCs

Derivation of human iPSCs from SCD patient cells is illustrated in Fig. 1b.

SCD Human Dermal Fibroblasts (HDFs) were cultured in DMEM supplemented with 10% FBS, and SCD keratinocytes were cultured in Dermal Life K Medium Complete Kit (Lifeline). For episomal plasmid reprogramming, 5 μg of pCLEB-R6 were electroporated into 5 × 10^5^ SCD HDFs using a Lonza Nucleofector (NHDF Nucleofector^®^ Kit, Program U-23). For rCLAE-R6 hybrid vector reprogramming, the vector was added to suspended single cells which were then gently shaken for 1 hr before seeding into a 6-well plate. The cells were trypsinized at 7 days post-transduction, and were replated onto CF1 murine embryonic fibroblasts (MEFs) in 100 mm dishes (Corning). The culture medium was replaced the next day with mTeSR1 medium (Stem Cell Technologies) supplemented with 0.25 mM sodium butyrate^38^ for fibroblast reprogramming or without sodium butyrate for keratinocyte reprogramming. Colonies with an appearance similar to human ESCs were picked between days 18–28 post-transduction; iPSCs reprogrammed from keratinocytes were usually picked one week earlier than iPSCs derived from fibroblasts.

Established SCD iPSC colonies were characterized by (1) PCR to examine residual reprogramming vectors (Fig. 1d), (2) teratoma formation following transplantation under the kidney capsule of immunodeficient (NSG) mice (Fig. 1f), and (3) karyotyping (Cell Line Genetics, Supplementary Fig. 2c).

### CRISPR/Cas Vector Construction

To construct CRISPR/Cas plasmids for *HBB* targeting, sgRNA oligos were synthesized by IDT and cloned into pX330 (Addgene: plasmid #42230) and pX335 (Addgene: plasmid #42335), respectively, following the Zhang lab protocol (https://www.addgene.org/crispr/zhang/).

To construct CRISPR/Cas double nicking plasmids for *HBB* targeting, human 7SK promoter-driven truncated sgRNA sequences^49^ were synthesized by IDT and cloned into the KpnI site of one sgRNA-bearing pX335 vector by Gibson Assembly (NEB).

The CRISPR/Cas components of pX330-HBB-T2 were transferred to the shuttle vector pM7.7 via PciI and NotI sites to derive pM7.7-330- HBB-T2.

### CRISPR/Cas sgRNA Testing

Human embryonic kidney (HEK) 293A cells (Invitrogen) were maintained in Dulbecco’s modified Eagle’s Medium (DMEM) supplemented with 10% fetal bovine serum (HyClone), 2 mM L-Glutamine (Corning), and 1X penicillin/streptomycin solution (Corning) at 37 °C with 5% CO_2_ incubation.

Two hundred thousand 293A cells were seeded into 12-well plates (Corning) one day prior to transfection. The cells were transfected with 1 μg CRISPR/Cas plasmid and 2.5 μl Lipofectamine 2000 (Invitrogen) following the manufacturer’s instructions.

Cells were harvested and genomic DNA was extracted 3 days post-transfection using 50 μl of 1X prepGEM (prepGEM Tissue Kit; ZyGEM) according to the manufacturer’s instructions. Two microliters of the DNA extract was added to a 25 μl PCR mix containing 2.5 μl of 10x LA Buffer, 0.25 μl of LA Taq (Takara), 2 ul of 2.5 mM dNTPs, and 0.25 ul of 20 uM each primer R135 and R136. Reactions were incubated at 95 °C for 5 min followed by 35 cycles of 95 °C 30 sec; 60 °C 30 sec, and 72 °C 70 sec. PCR products were analyzed by the SURVEYOR assay (Transgenomics) following the manufacturer’s instructions and using 4–20% TBE protein gels (Bio-Rad). Gels were stained with ethidium bromide (EB) for 30 min and imaged using a Gel Doc gel imaging system (Bio-Rad).

### Adenoviral CRISPR/Cas Vector Construction

The multiple cloning site (MCS) of pM7 was transferred to pAd/PL-DEST by LR recombination (Invitrogen) to derive pAd/PL-M7 which has a PI-SceI site between *att*B1 and *att*B2. The CRISPR/Cas components of pM7.7-330- HBB-T2 were transferred to pAd/PL-M7 to derive pAd-T2 by subcloning into the PI-SceI site.

### Recombinant Adenovirus Production

Ten micrograms of recombinant adenoviral plasmid were digested with PacI and packaged into rAd-T2 following the manufacturer’s instructions (Invitrogen).

The titer of rAd-T2 was determined by the end-point dilution assay.

### Recombinant HDAd/EBV Hybrid Virus Production

Ten micrograms of recombinant adenoviral plasmids were digested with AscI and packaged as an HDAdV as described^50^.

### CRISPR/Cas-enhanced Gene Targeting in Human iPSCs with G418 Selection

iPSCs were dissociated with accutase (Stem Cell Technology) for 5 min to generate a single cell suspension. Two million cells were washed with DMEM/F12 medium and resuspended in 100 ul Nucleofector Solution. Two and a half micrograms of CRISPR/Cas plasmid and 2.5 μg HindIII-linearized correction template PJ2.2 (derived from PJ2^51^) were added to Nucleofector Solution containing the iPSCs and the suspension was gently mixed before electroporation (Human Stem Cell Nucleofector Kit 1, program A-23, Lonza). Electroporated cells were plated into Matrigel coated 6-well plates in mTeSR1 medium supplemented with 10 μM ROCK inhibitor Y-27632 (EMD Millipore)and 25 μg/ml G418 was added to the medium 24 hr later and maintained for 2 weeks. Colonies were picked and expanded for continuous culture and genotyping. For genotyping, a 5′ primer set (R153 + R148) and a 3′ primer set (R146+R173) were used. To remove the neomycin resistance marker, iPSCs were infected with a Cre-expression recombinant adenoviral vector (rAd-Cre-IE^37^) and analyzed by PCR for marker deletion with the primer set R215+R216.

### Isolation of Gene-Corrected iPSCs using Sib-selection

Isolation of gene-corrected iPSCs by sib-selection is illustrated in Fig. 2a.

Two millions single iPSCs were prepared and electroporated with 5 μg pM7.7-330-HBB-T2 and 1 μg ssODN-T2 (Human Stem Cell Nucleofector Kit 1, program A-23, Lonza). The electroporated cells were plated into a Matrigel-coated 96-well plate (Corning) using a multichannel micropipetter. G418 was added to the medium at 25 μg/ml 8 hr post-transfection and maintained for 40 hr for temporary selection of transfected cells using the neomycin resistance gene in the backbone of vector pM7.7.

When iPSCs were 80–90% confluent, the cells were washed with DMEM/F12 medium and split into two 96-well plates at a 3:1 density ratio. The plate with more cells was used for genomic DNA extraction and the other plate for continuous iPSC culture. Three to four hr after splitting or the next day, the high-cell-density plate was washed with 100 μl/well PBS and genomic DNA was extracted with 25 μl/well prepGEM.

For the detection of T to A point mutation corrections, ddPCR components were mixed as follows: 11 μl of 2X ddPCR Supermix for Probes (Bio-Rad), 1 μl of 20 μM primer R196, 1 μl of 20 μM primer R197, 1 μl of 5 μM HBB-wt-FAM, 1 μl of 5 μM HBB-sk-VIC, 2 μl of genomic DNA lysate, and 5 μl of ddH_2_O (22 μl total rxn volume). Twenty microliters of the rxn were transferred to DG8 cartridges (Bio-Rad) and droplets were generated with a QX200 Droplet Generator (Bio-Rad). The droplets were transferred into a 96-well twin tec PCR plate (Eppendorf), sealed with a PX1 PCR Plate Sealer (Bio-Rad), and PCR was performed in a C1000 Touch Thermal Cycler (Bio-Rad). The thermal cycling program conducted was: Step 1: 95 °C 10 min; Step 2: 95 °C 30 sec; Step 3: 55 °C 1 min; repeat steps 2–3 39 times; Step 4: 98 °C 10 min; Step 5: 8 °C hold. Following PCR, the droplets were analyzed using a QX200 Droplet Reader (Bio-Rad) with the “absolute quantification” option.

The wells that contained over 5% β^A^ allele frequency were used for single colony isolation. Cells from these wells were plated at densities of 10–30 cells/well into 96-well plates (the cell number required to seed for single colonies differ among iPSC lines and are tested by limiting dilution in advance). The cells were cultured in mTeSR1 medium supplemented with 10 μM Y-27632 for 6 days, and then in mTeSR without Y-27632 for another 4 days. The wells containing single colonies were then split into two wells, one to be used for genomic DNA extraction for ddPCR and Sanger sequencing.

### Gene Targeting with CRISPR/Cas Delivered by Adenoviral Vector

Single iPSCs were seeded at 5 × 10^5^ cells/well into Matrigel-coated 6-well plates and cultured in mTeSR1 supplemented with 10 μM Y-27632 one day prior to infection. The next day, the CRISPR/Cas expression recombinant adenovirus was added directly into the medium and incubated at 37 °C for 1 hr. Subsequently, single iPSCs were prepared and 20 μg of correction ssODN was electroporated into the cells as described above. Genomic DNA was extracted with prepGEM and analyzed by ddPCR 72 hr later.

### Whole-Genome Sequencing and Analysis

WGS was performed as previously described^52^. The WGS data can be accessed at the NCBI SRA database with the accession number SRP075463.

## Additional Information

**How to cite this article**: Li, C. *et al*. Novel HDAd/EBV Reprogramming Vector and Highly Efficient Ad/CRISPR-Cas Sickle Cell Disease Gene Correction. *Sci. Rep.* **6**, 30422; doi: 10.1038/srep30422 (2016).

## Supplementary Material

Supplementary Information

## Figures and Tables

**Table 1:** Identification of Potential Off-Target Sites.

Examined sites	# of potential off-target sites	# of off-target site found in 11-B4D8	# of off-target site found in 12-G3B6
0 mismatch	1	0	0
1 base mismatch, potential off-target sites	0	0	0
2 base mismatch, potential off-target sites	0	0	0
3 base mismatch, potential off-target sites	9	0	0
4 base mismatch, potential off-target sites	119	0	0
5 base mismatch, potential off-target sites	1348	0	0

Potential off-target sites were identified by aligning the CRISPR/Cas9 guide sequence to the hg19 reference genome using EMBOSS fuzznuc software (v.6.6.0.0) and allowing for a maximum of five mismatches.

11-B4D8 and 12-G3B6 are corrected iPSC lines derived from two SCD patients.
