# Supplementary material for: Novel HDAd/EBV Reprogramming Vector and Highly Efficient Ad/CRISPR-Cas Sickle Cell Disease Gene Correction
Source: Sci Rep. 2016 Jul 27;6:30422. doi: 10.1038/srep30422 (PMC4961958; doi:10.1038/srep30422)
Supplement: Supplementary Information [file srep30422-s1.pdf]

Novel HDAd/EBV Reprogramming Vector and Highly Efficient Ad/CRISPR-Cas Sickle Cell Disease Gene Correction

Chao Li[1,2,*], Lei Ding[1,2,*], Chiao-Wang Sun[1,2], Li-Chen Wu[1,2], Dewang Zhou[1,2], Kevin M Pawlik[1,2], Alireza Khodadadi-Jamayran[2], Erik Westin[1,2], Frederick D. Goldman[2,3], and Tim M Townes[1,2]

[1]Department of Biochemistry and Molecular Genetics
[2]UAB Stem Cell Institute
[3]Department of Pediatrics, Division of Hematology/Oncology
Schools of Medicine and Dentistry, University of Alabama at Birmingham, Birmingham, AL 35294, USA

[*]These authors contributed equally to this work.

Correspondence to: Tim M Townes | ttownes@uab.edu

SUPPLEMENTARY INFORMATION

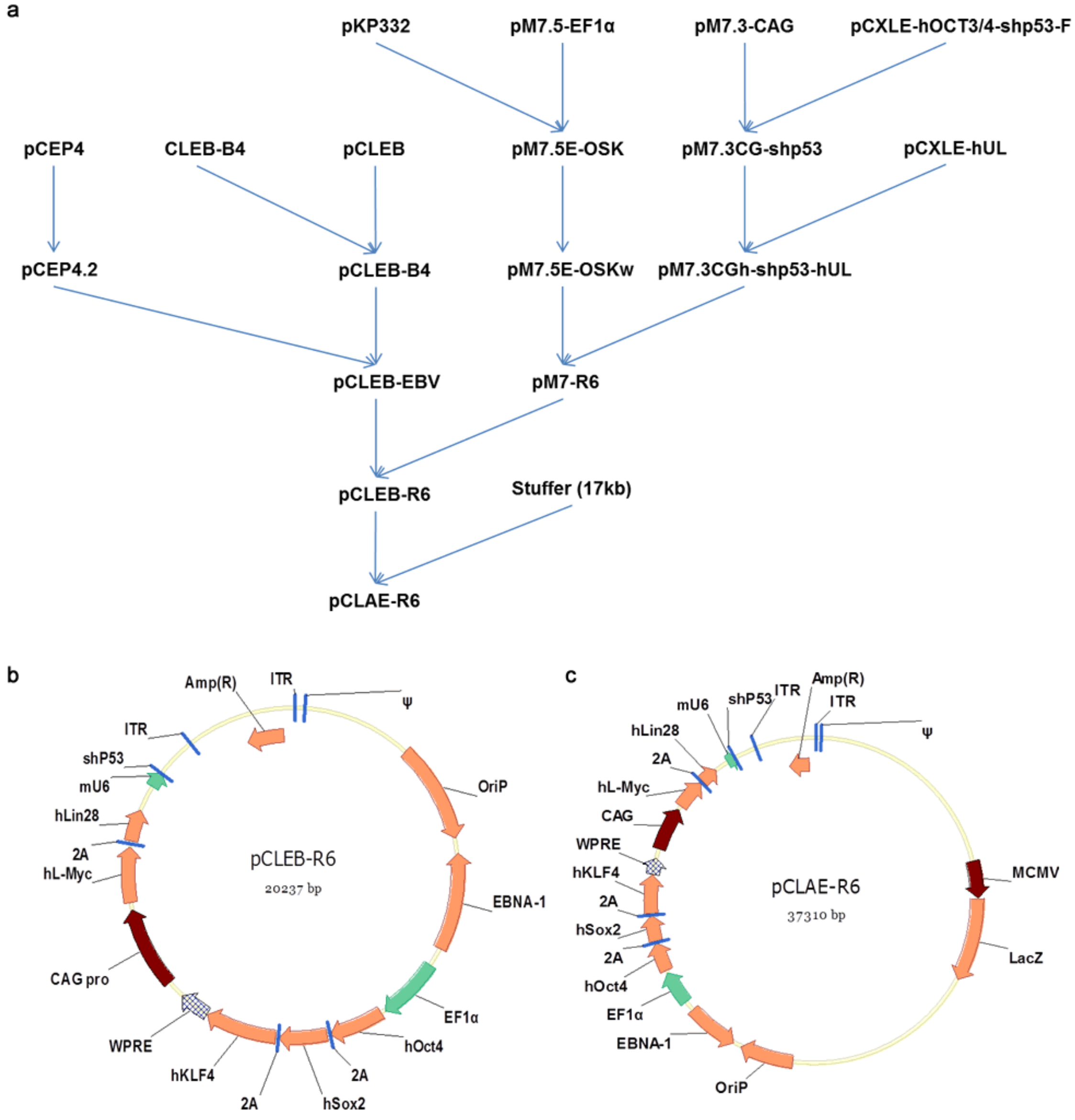

Supplementary Figure 1. HDAd/EBV Hybrid Reprogramming Vector Construction

a. Schematic representation of the 6-factor HDAd/EBV hybrid reprogramming vector construction.

b. Map of the 6-factor episomal reprogramming vector pCLEB-R6.

c. Map of the 6-factor HDAd/EBV hybrid reprogramming vector pCLAE-R6

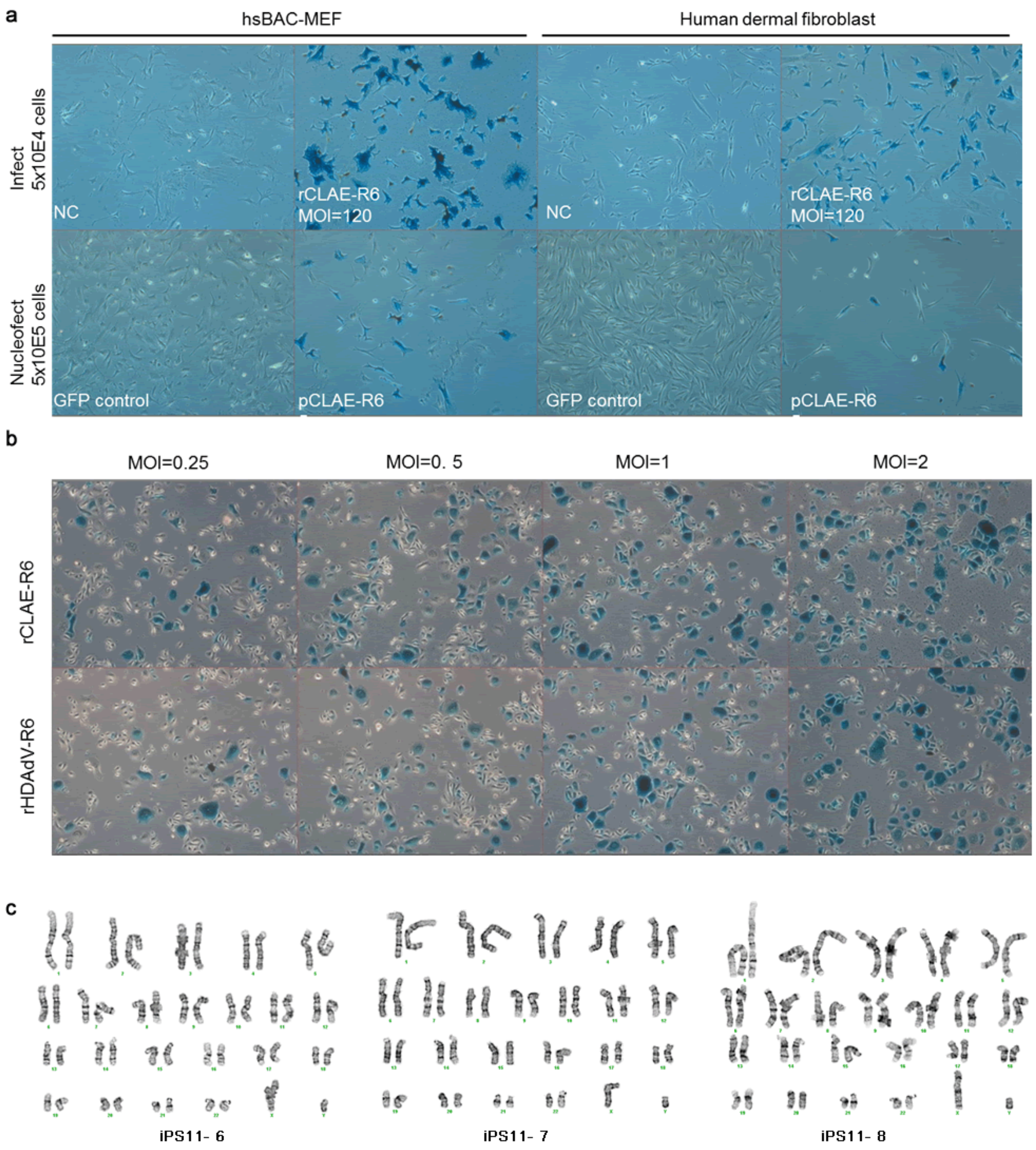

Supplementary Figure 2. Efficient Transduction of Fibroblasts and Keratinocytes with rCLAE-R6

a. Transduction efficiency and survival rate comparison of rCLAE-R6 infection and pCLAE-R6 electroporation to fibroblasts. Both mouse embryonic fibroblast, hsBAC-MEF, and human dermal fibroblast (BJ cell line) were used. $5x10^4$ cells

were infected with rCLAE-R6, while $5x10^5$ cells were eletroporated with pCLAE-R6. X-Gal staining was performed 48 hr post-transduction.

b. Transduction optimization of rCLAE-R6 and rHDAdV-R6 to sickle patient keratinocytes. MOIs 0.25, 0.5, 1, 2 were tested.

c. Karyotype analysis was performed on G-banded metaphase cells from iPS11-6, 7, 8. All three lines demonstrated apparently normal karyotypes and expected chromosome counts.

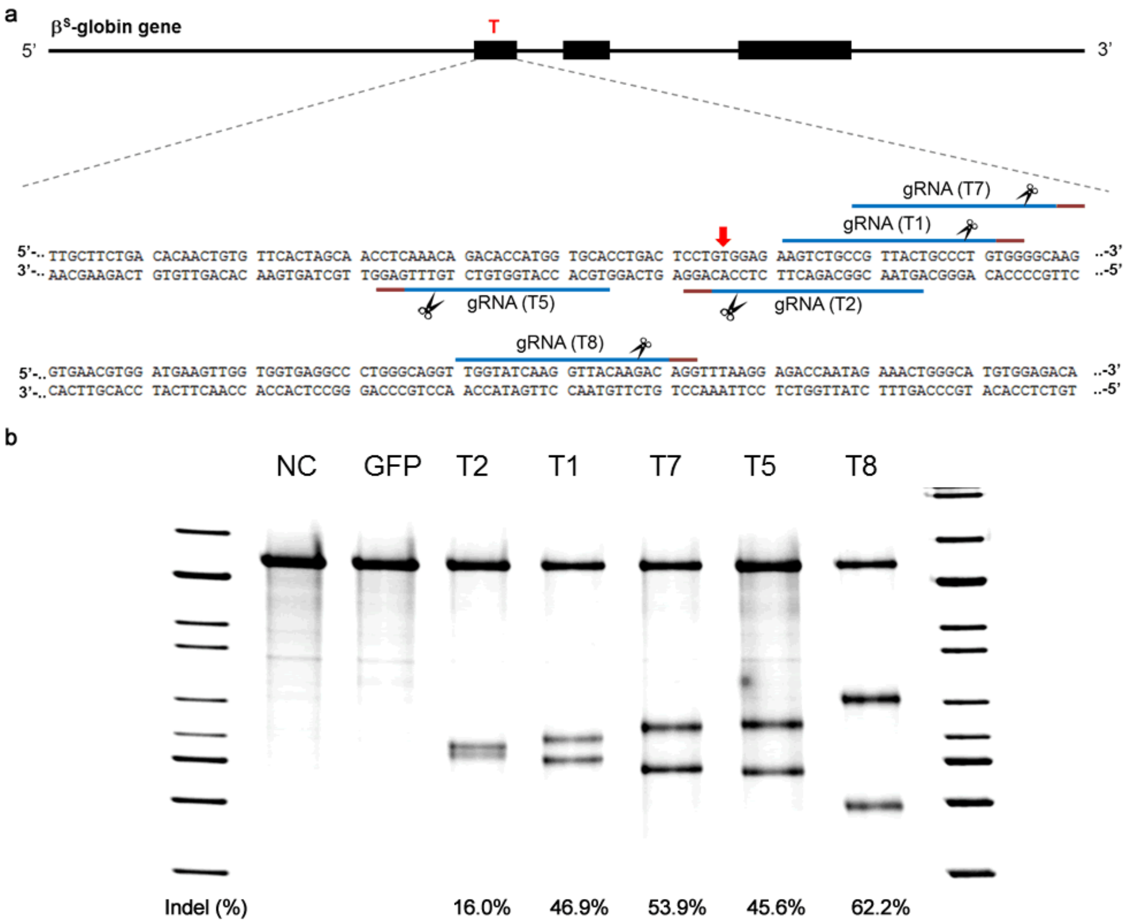

Supplementary Figure 3. *HBB* sgRNA Design and Efficiency Verification

a. Schematic of the human *HBB* locus showing the location of five protospacers indicated by blue lines with corresponding PAMs in red lines.

b. Surveyor assay comparing the efficacy of Cas9-mediated cleavage at five protospacers in the human *HBB* locus in 293A cells. The bands were quantified with Image Lab 3.0 (Bio-Rad).

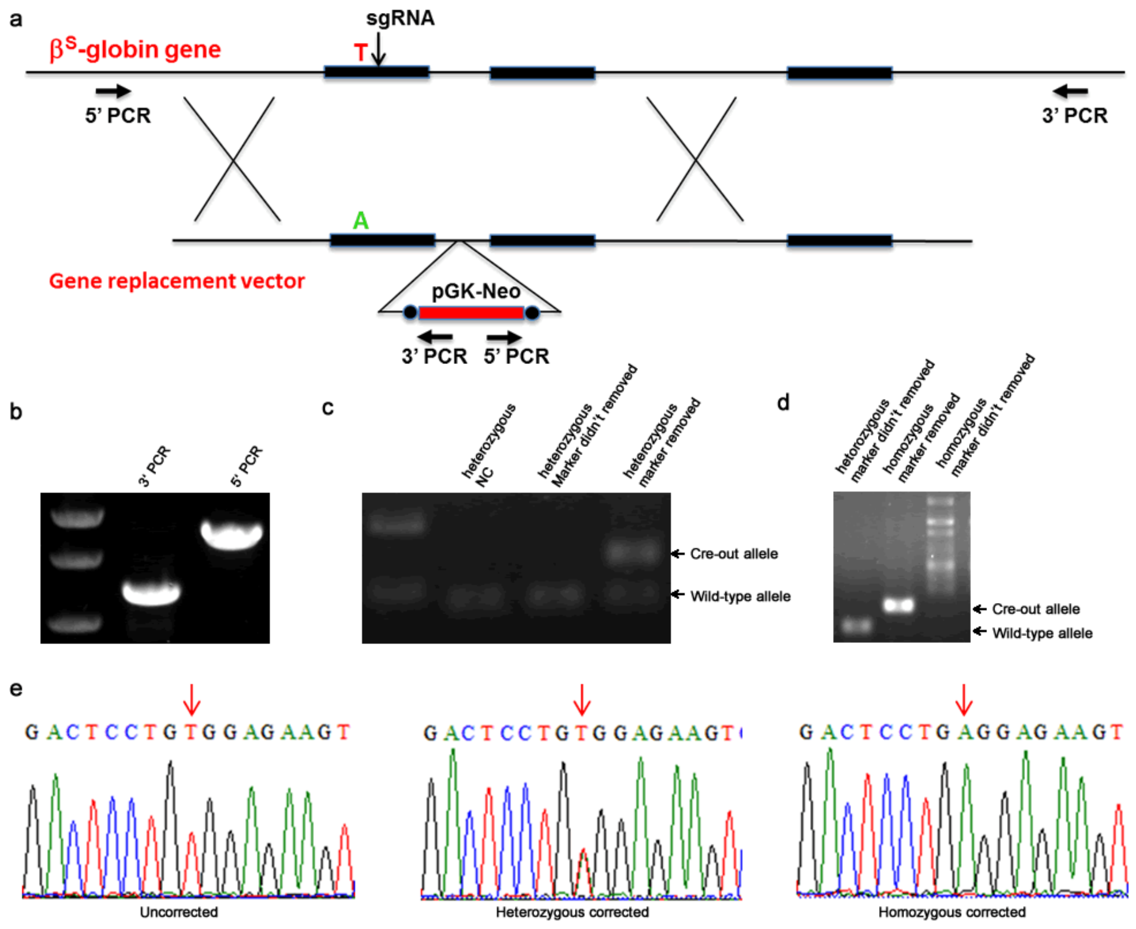

Supplementary Figure 4. CRISPR/Cas Enhanced Gene Correction of Sickle Mutation in Patient Derived iPSCs via Antibiotic Selection.

- **a.** Schematic representation of CRISPR/Cas *HBB* gene correction with a DNA correction template. The primers for PCR are shown as arrows. pGK promoter-driven neomycin resistance gene was used for positive selection.
- **b.** PCR verification of G418 resistant colonies; primers for correct 5'- and 3'-end gene targeting are indicated by black arrows in panel a.
- **c.** PCR analysis of corrected iPSC clones treated with the Cre-expressing adenovirus (rAd-Cre-IE). (left) PCR analysis for selection marker removal of heterozygous corrected iPSC lines. (right) PCR screen for selection marker removal from homozygously corrected iPSC lines.

d. Sanger sequencing results of PCR amplicons from uncorrected SCD iPSC line (left), a heterozygously corrected line (middle), and homozygously corrected line (right). The heterozygous clone was corrected with wtCas9 + (T8+T2), and the homozygous clone was corrected with wtCas9 + T2.

Supplementary Table 1.Summary of Gene Targeting Efficiency of Different Cas9 and sgRNA Combinations

Cas9 and sgRNA combination	Examined colony #	PCR verified colony #	Sequencing verified colony #	Heterozygous corrected colony #	Homozygous corrected colony #	Corrected w/ indel colony #	Percentage of correction w/o indel
wtCas9 + HBB-T1	30	8	8	8	0	6	25%
wtCas9 + HBB-T2	33	4	3	2	1	0	100%
nCas9 + HBB-T1	21	1	1	1	0	0	100%
nCas9 + HBB-(T1+T2)	12	0	0	0	0	n.a.	n.a.
nCas9 + HBB-(T1+T5)	12	1	1	1	0	0	100%
nCas9 + HBB-(T8+T2)	51	4	4	4	0	0	100%
nCas9 + HBB-(T8+T5)	25	0	0	0	0	n.a.	n.a.

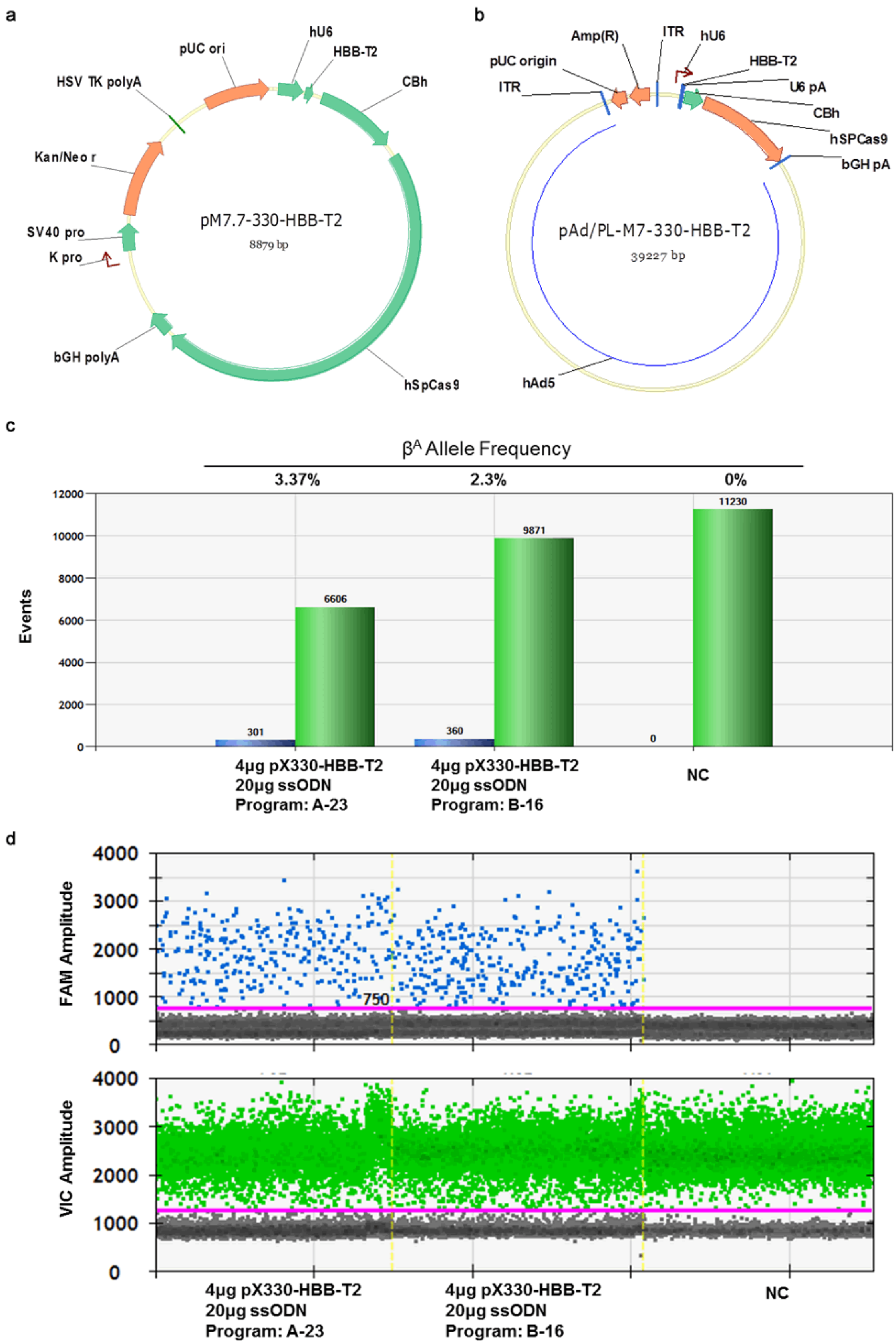
a
pUC ori
hU6
HBB-T2
CBh
HSV TK polyA
Kan/Neo r
SV40 pro
K pro
bGH polyA
hSpCas9
pM7.7-330-HBB-T2
8879 bp
b
Amp(R)
ITR
hU6
pUC origin
HBB-T2
ITR
U6 pA
CBh
hSPCas9
bGH pA
hAd5
pAd/PL-M7-330-HBB-T2
39227 bp
c
βA Allele Frequency
3.37%
2.3%
0%
Events
12000
10000
8000
6000
4000
2000
0
301
6606
360
9871
0
11230
4µg pX330-HBB-T2
20µg ssODN
Program: A-23
4µg pX330-HBB-T2
20µg ssODN
Program: B-16
NC
d
FAM Amplitude
4000
3000
2000
1000
0
750
VIC Amplitude
4000
3000
2000
1000
0
4µg pX330-HBB-T2
20µg ssODN
Program: A-23
4µg pX330-HBB-T2
20µg ssODN
Program: B-16
NC

Supplementary Figure 5. Improved Correction Efficiency with Increased Amount of ssODN

a. Map of pM7.7-330-HBB-T2.

b. Map of pAd/PL-M7-330-HBB-T2.

c. *HBB* corrected allele frequency as measured by ddPCR in pooled SCD iPSCs 72 hrs post-electroporated with pM7.7-330-HBB-T2 and ssODN. Green bar: β^S–positive droplets; Blue bar: β^A–positive droplets.

d. *HBB* allele frequency as measured by ddPCR and represents as raw droplet data in pooled SCD iPSCs 72 hrs post-electroporation with pM7.7-330-HBB-T2 and ssODN. (top) FAM droplet data represents the β^A allele, and (bottom) VIC droplet data represents the β^S allele.

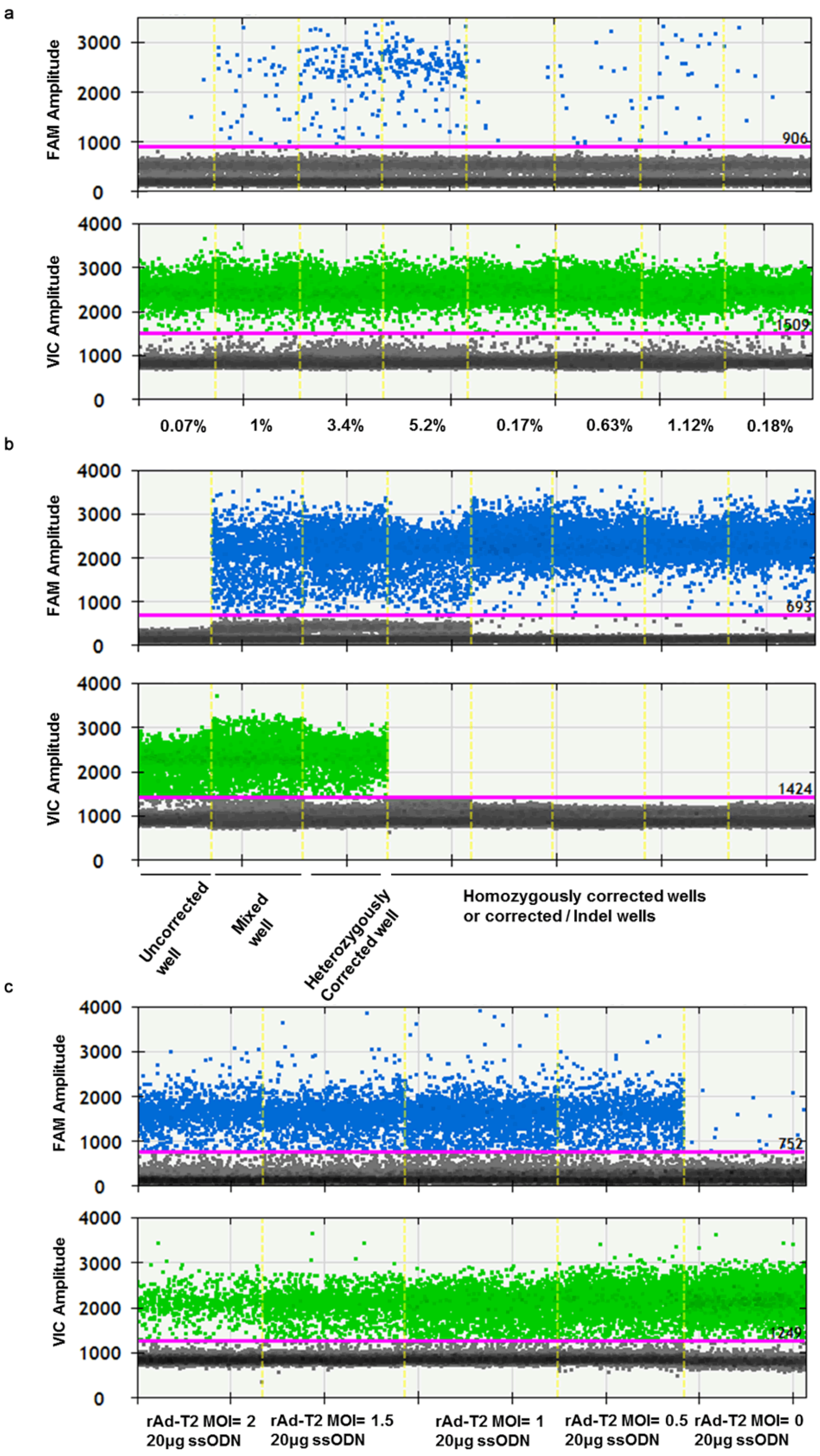
a
FAM Amplitude
VIC Amplitude
906
1509
0.07%
1%
3.4%
5.2%
0.17%
0.63%
1.12%
0.18%
b
FAM Amplitude
VIC Amplitude
693
1424
Uncorrected well
Mixed well
Heterozygously Corrected well
Homozygously corrected wells
or corrected / Indel wells
c
FAM Amplitude
VIC Amplitude
752
1249
rAd-T2 MOI= 2
20µg ssODN
rAd-T2 MOI= 1.5
20µg ssODN
rAd-T2 MOI= 1
20µg ssODN
rAd-T2 MOI= 0.5
20µg ssODN
rAd-T2 MOI= 0
20µg ssODN

Supplementary Figure 6. Raw Droplet Data of Sib-selection Based Correction.

a. Raw droplet data from the first sib-selection following pM7.7-330-HBB-T2 + ssODN electroporation into SCD iPSCs. (top) FAM droplet data represents the β^A allele, and (bottom) VIC droplet data represents the β^S allele.

b. Raw droplet data from the second sib-selection. (top) FAM droplet data represents the β^A allele, and (bottom) VIC droplet data represents the β^S allele.

c. Raw droplet data rom the rAd-T2 infection + ssODN electroporation into SCD iPSCs. (top) FAM droplet data represents the β^A allele, and (bottom) VIC droplet data represents the β^S allele.

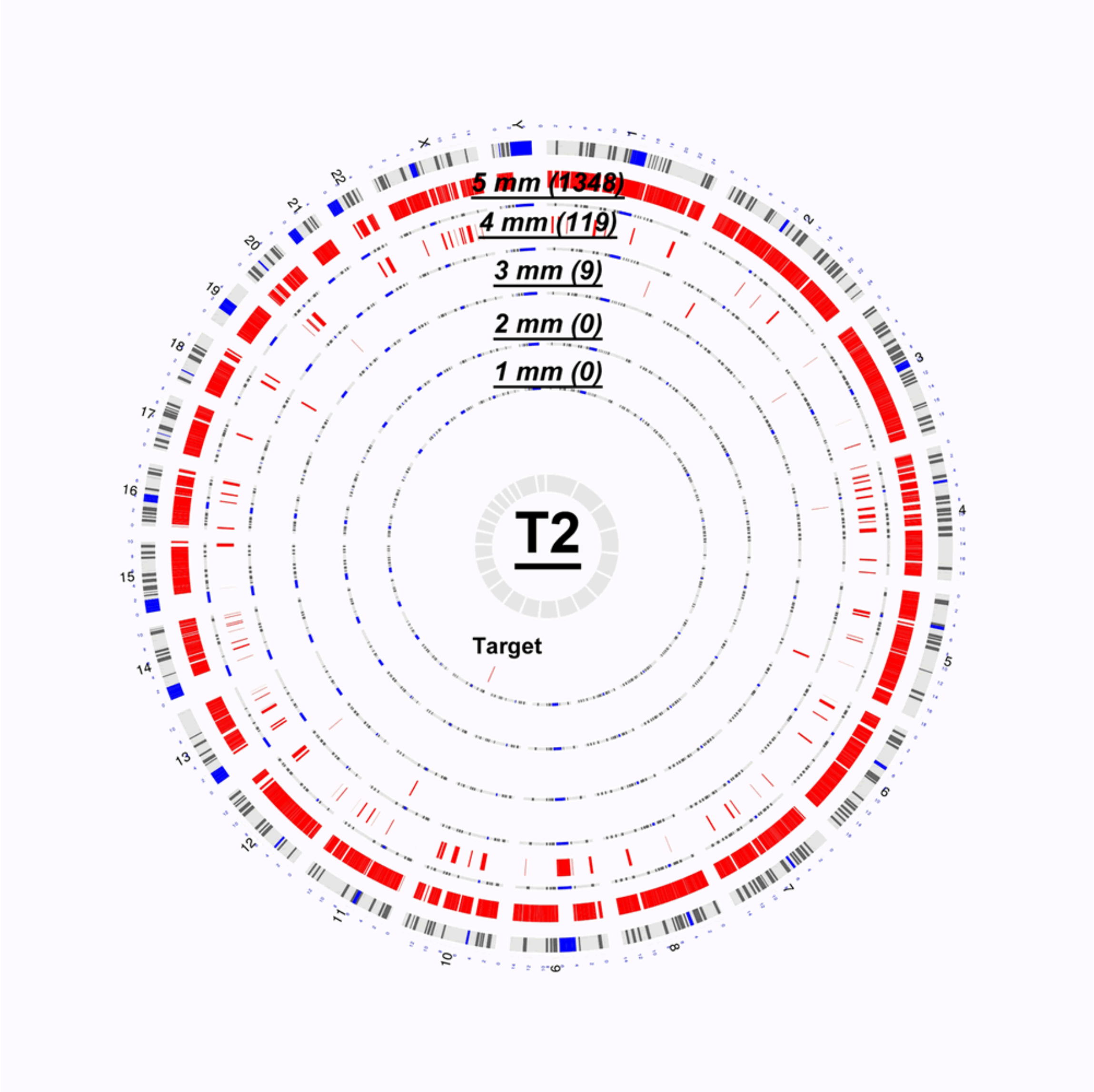

Supplementary Figure 7. Genome Distribution of Potential Off-target Sites. Potential off-target sites were identified by aligning the CRISPR/Cas9 guide sequences to the hg19 reference genome using EMBOSS fuzznuc software (v6.6.0.0) (Rice et al., 2000) and allowing for a maximum of three mismatches. Potential off-target sites are displayed using OmiCircos software. mm: mismatch.

(http://bioconductor.org/packages/release/bioc/html/OmicCircos.html).

Supplementary Table 2

Primers List	
Reprogramming vector (pCLAE-R6) construction	
CLEB-B4	ccgtagatctgcgctcgagtctagctagcgaatttacgtagaagttcctattctctagaaagtata ggaacttctgccgcagatccgggggcaatgaaaaagcctgaactcaccgcgacgtctgtcg agaagtttctgatcgaaaagttccctcttcaaggtcaccggatcctaactataacggtcctaag gtagcgaaacctgggaggcagaggttgatctatgtcgggtgcggagaaagaggtaatgaa atggcactctgcagccccggaagaaatatatttgcatgtctttagttctatgatgacacaaaccc cgcccagcgtcttgtcattggcgaattcgaacacgcagatgcagtcggggcggcgcggtccg aggtccacttcgcatattaaggtgacgcgtgtggcctcgaacaccgagcggtcgacgaagtt cctattctctagaaagtataggaacttccaattgatttaaatgcggccgcactagtca
PCR for residual reprogramming vectors	
OriP-f	TTCGCCTGCTTCTTCATTCTCC
OriP-r	GGTTCACCTGTCTTGGTCCCT
CRISPR/Cas vector construction	
HBB-T1-f	caccgGTCTGCCGTTACTGCCCTG
HBB-T1-r	aaacCAGGGCAGTAACGGCAGACc
HBB-T2-f	caccgTAACGGCAGACTTCTCCaC
HBB-T2-r	aaacGaGGAGAAGTCTGCCGTTAc
HBB-T5-f	caccgGCACCATGGTGTCTGTTTG
HBB-T5-r	aaacCAAACAGACACCATGGTGCc
HBB-T7-f	caccgGTTACTGCCCTGTGGGGCA
HBB-T7-r	aaacTGCCCCACAGGGCAGTAACc
HBB-T8-f	caccgGGTATCAAGGTTACAAGAC
HBB-T8-r	aaacGTCTTGTAACCTTGATACCc
Genotyping for gene targeting with selection resistant marker	
R153	CCTGCACCTGCTGTGGCATCCA
R148	GCTCCAGACTGCCTTGGGAAAAGC
R146	GATCAGCAGCCTCTGTTCCACA
R173	TAAATGCACTGACCTCCCACATTC
Primers to screen selection marker removal	
R215	CAATAGAAACTGGGCATGTGGA
R216	GTGGGAAAATAGACCAATAGGCA
Correction template for sib-selection based gene targeting	
ssODN	CTTGCCCCACAGGGCAGTAACGGCAGACTTCTCCTCAGGAGTC AGGTGCACCATGGTGTCTGTTTGAGGT
Taqman probes for ddPCR	
HBB-wt-FAM	FAM-TGACTCCTGAGGAGAA-MGB
HBB-sk-VIC	VIC-ACTCCTGTGGAGAAG-MGB
Primers for ddPCR	

R196	CAGAGCCATCTATTGCTTACATTTG
R197	GGCCTCACCACCAACTTCAT
